# Supplementary material for: Robust functional ultrasound imaging in the awake and behaving brain: A systematic framework for motion artifact removal
Source: Imaging Neurosci (Camb). 2026 Apr 7;4:IMAG.a.1191. doi: 10.1162/IMAG.a.1191 (PMC13058852; doi:10.1162/IMAG.a.1191)
Supplement: Supplementary Material [file IMAG.a.1191_supp.pdf]

Supplementary Material

Robust functional ultrasound imaging in the awake and behaving brain: a systematic framework for motion artifact removal

Samuel Le Meur-Diebolt,[1,2,3] Felipe Cybis Pereira,[1,2] Jean-Charles Mariani,[3]
Andrea Kliewer,[4] Miguel Farinha-Ferreira,[3,5,6,7] Adrien Bertolo,[1,2]
Bruno-Félix Osmanski,[2] Zsolt Lenkei,[3†] Thomas Deffieux[1†*]

February 10, 2026

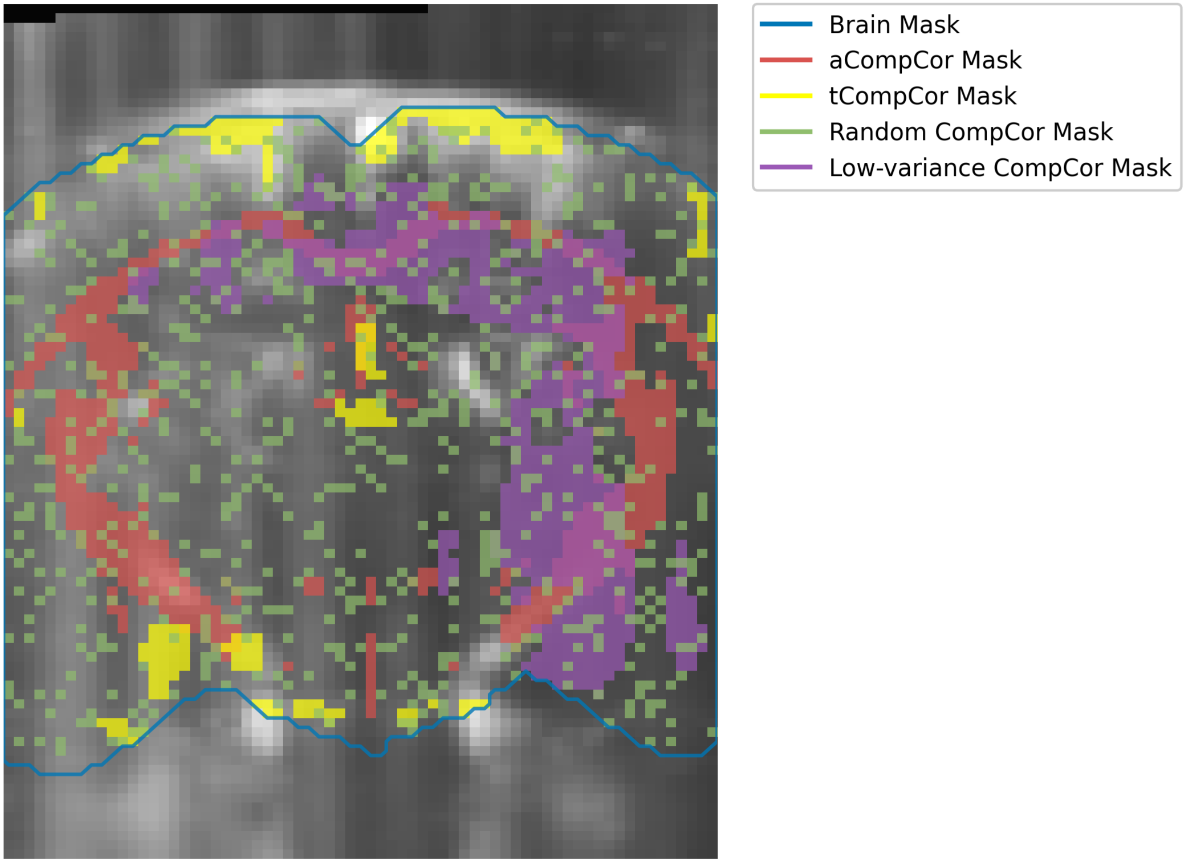

Figure S1: Spatial masks used for confound regression strategies. The aCompCor mask is defined anatomically, including white matter and CSF voxels derived from the Allen Mouse Brain CCFv3 atlas. The tCompCor mask includes the top 5% most variable voxels across time. The "low-variance" mask selects the subset of voxels with the lowest temporal standard deviation, matched in volume to the aCompCor mask. The "random" mask consists of a random selection of brain voxels, also matched in volume to the aCompCor mask. All masks are overlaid on an example power Doppler image from dataset 1.

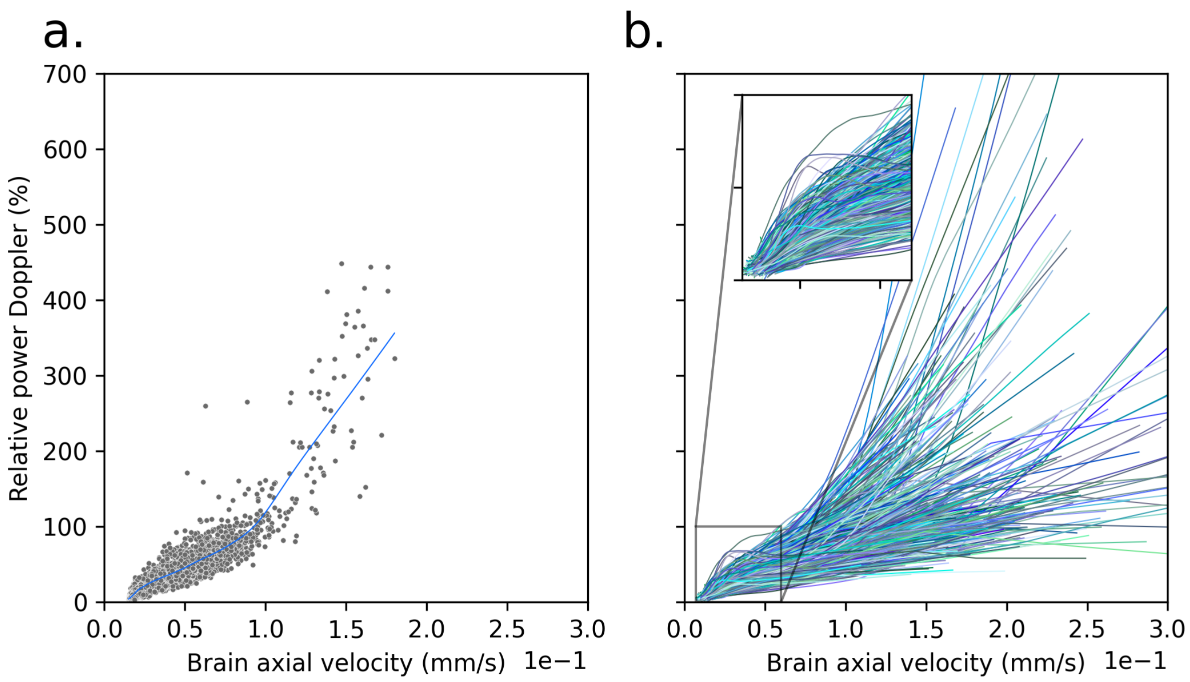

Figure S2: Power Doppler intensity is related to GV. (a.) The relative power Doppler signals averaged over brain voxels is plotted against the GV. A locally weighted scatterplot smoothing (LOWESS) curve is fitted on the data. (b.) LOWESS curves for all acquisitions from datasets 1 and 2 are shown, showing a non-linear dependence between power Doppler intensities and GV, even for small velocities.

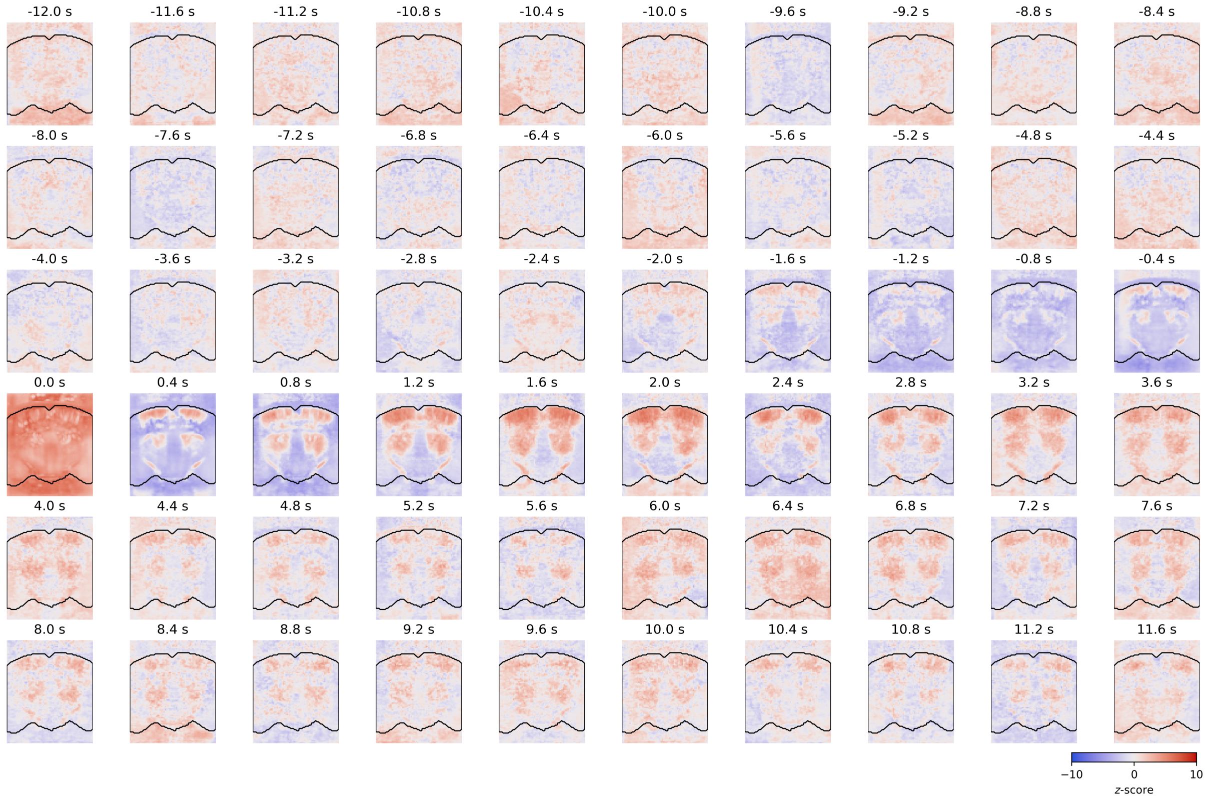

Figure S3: Brain movement induces both immediate and delayed effects on power Doppler signals. Maps are shown for all delays for the FIR model in Figure 3 of the main text.

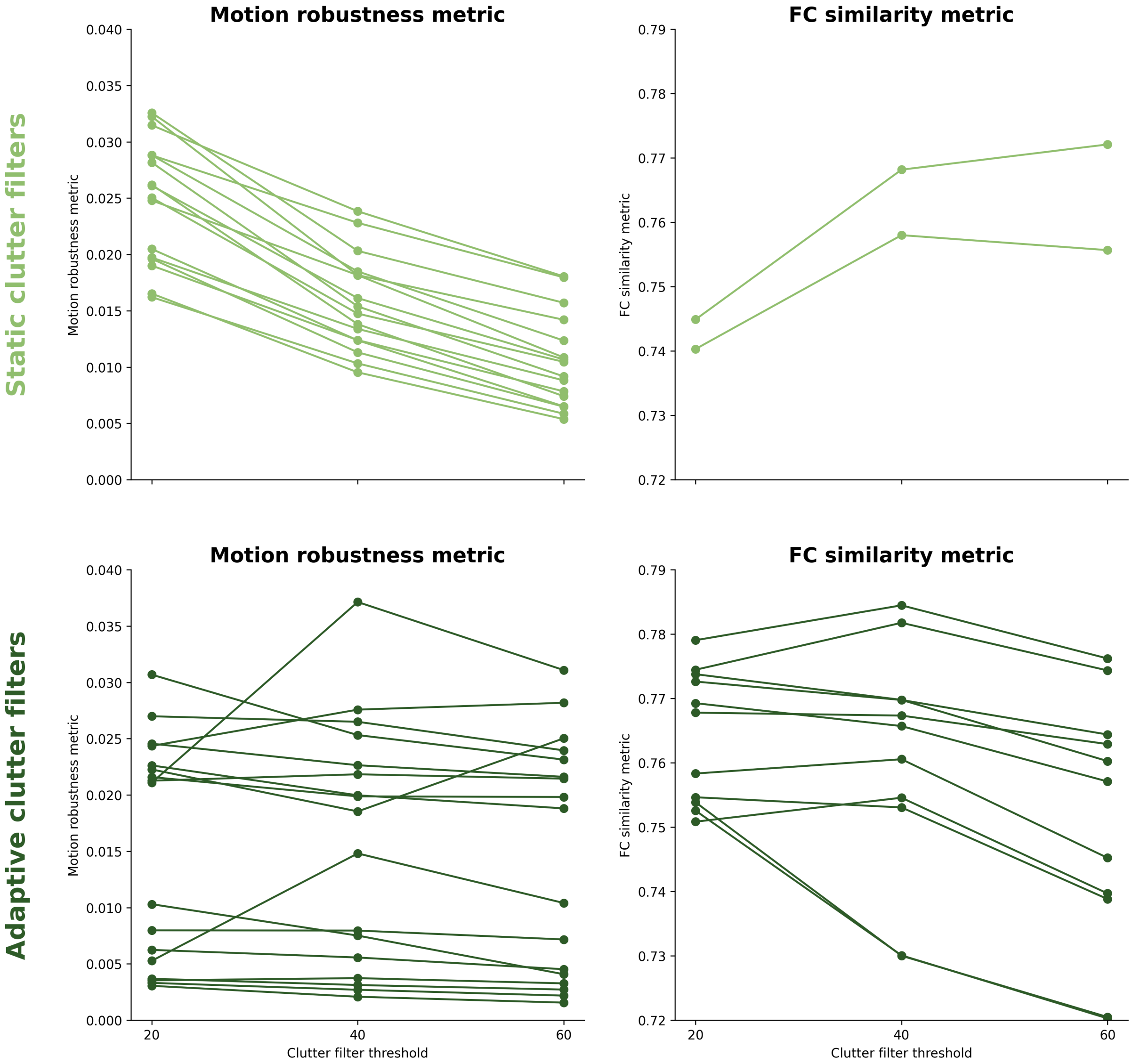

Figure S4: Metrics across clutter filter thresholds. Slope graphs show the motion robustness (left) and FC similarity (right) metrics across clutter filter thresholds. Metrics are shown using dataset 1 for all strategies that pass the corresponding metric threshold (either MSE < 0.025 or Dice > 0.75). Each row shows The top row shows the metrics for strategies using static SVD$_T$ clutter filters, while the bottom row shows the metrics for strategies using adaptive SVD$_T$ clutter filters.

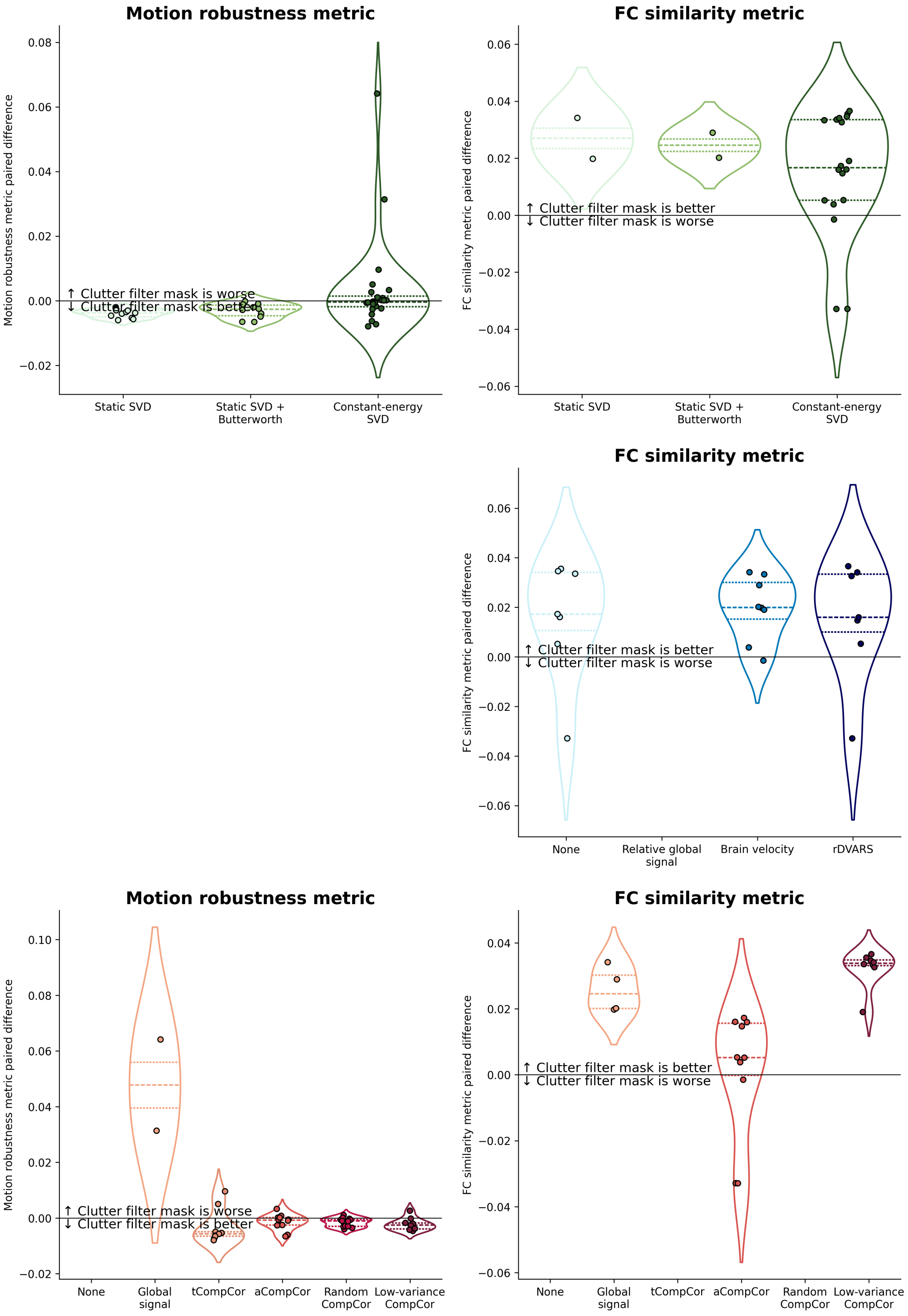

Figure S5: Paired metric differences for clutter filter masking strategies. Violin plots show the paired differences in motion robustness (left) and FC similarity (right) metrics between clutter filter masking strategies. Paired differences are computed using dataset 1 for all strategies that pass the corresponding metric threshold (either MSE < 0.025 or Dice > 0.75). Each row shows the same paired differences, colored by clutter filter, scrubbing metric, and confound regression method.

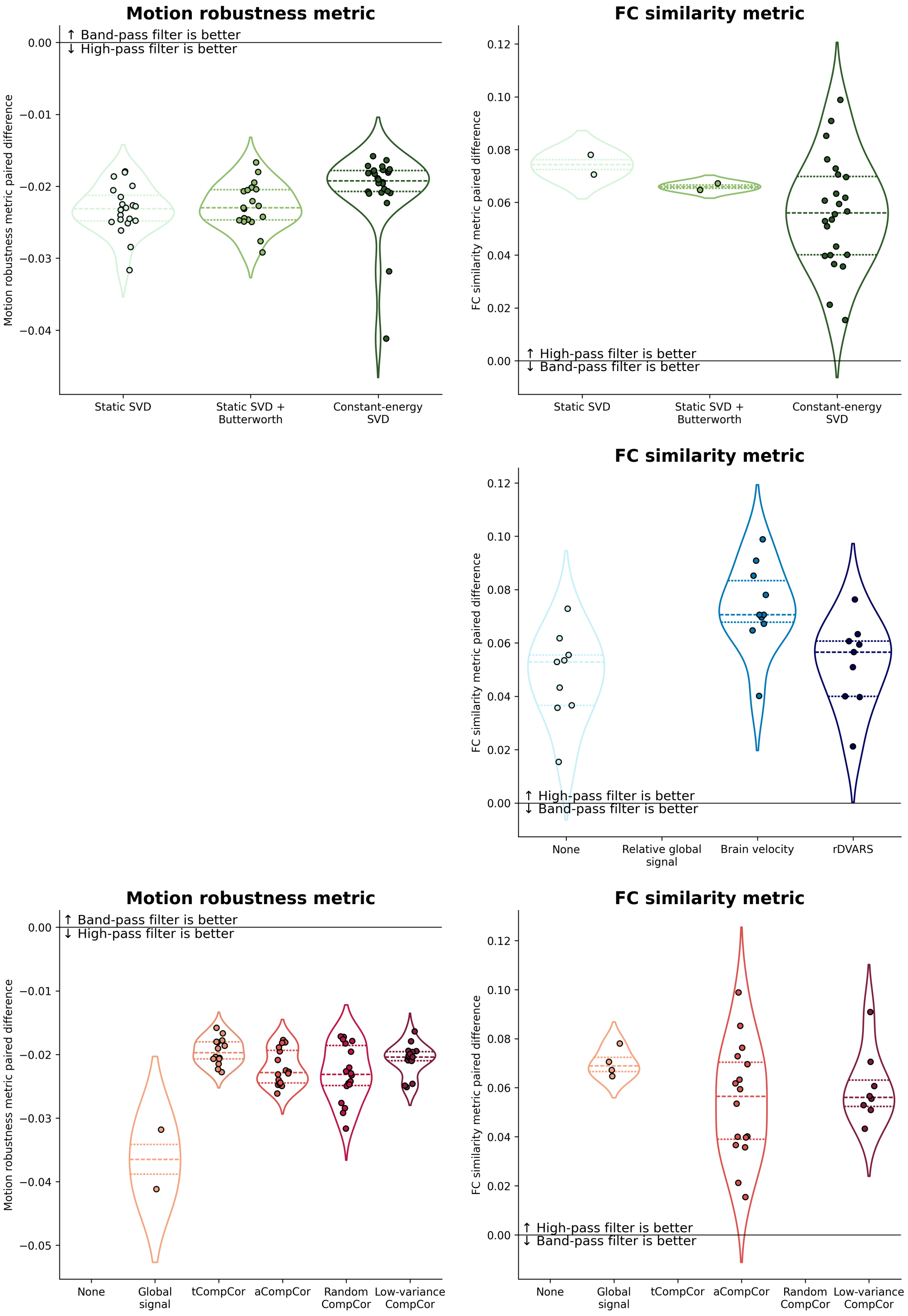

Figure S6: Paired metric differences for frequency filtering strategies. Violin plots show the paired differences in motion robustness (left) and FC similarity (right) metrics between high-pass and band-pass filtering strategies. Paired differences are computed using dataset 1 for all strategies that pass the corresponding metric threshold (either MSE < 0.025 or Dice > 0.75). Each row shows the same paired differences, colored by clutter filter, scrubbing metric, and confound regression method.

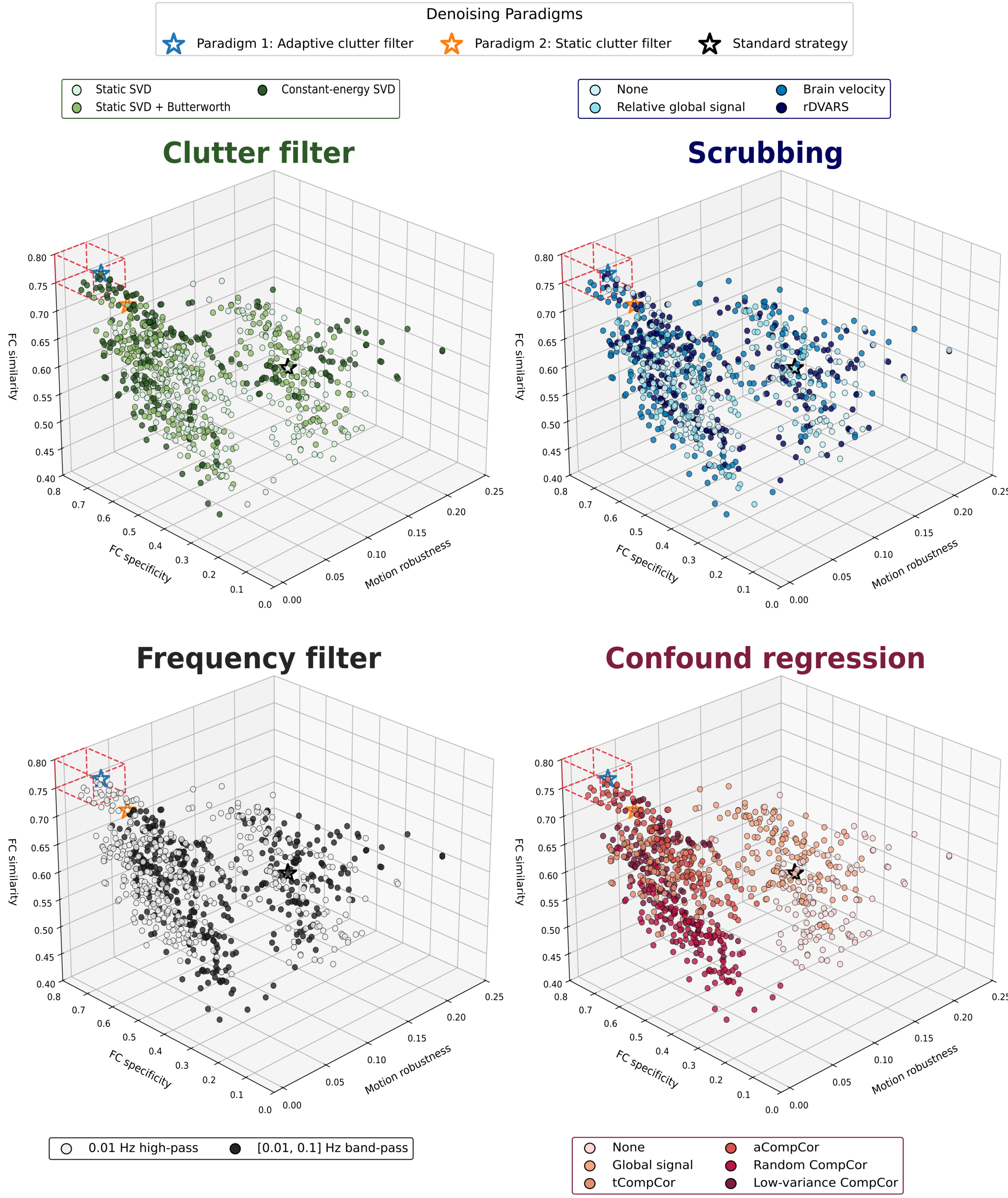

Figure S7: Optimized paradigms balance reliability and specificity. To address concerns regarding potential over-regularization, we benchmarked strategies using a third metric, termed "FC Specificity", adapted from the work of Desrosiers-Grégoire and colleagues and quantifying the inter-scan variability in functional connectivity is spatially distributed with respect to the "ground-truth" group-level patterns. The 3D scatter plots display the performance of all denoising strategies across all three metrics: motion robustness, FC similarity, and FC specificity. Only paradigm 1 satisfies high scores on all three axes (red dashed box), with paradigm 2 close behind.

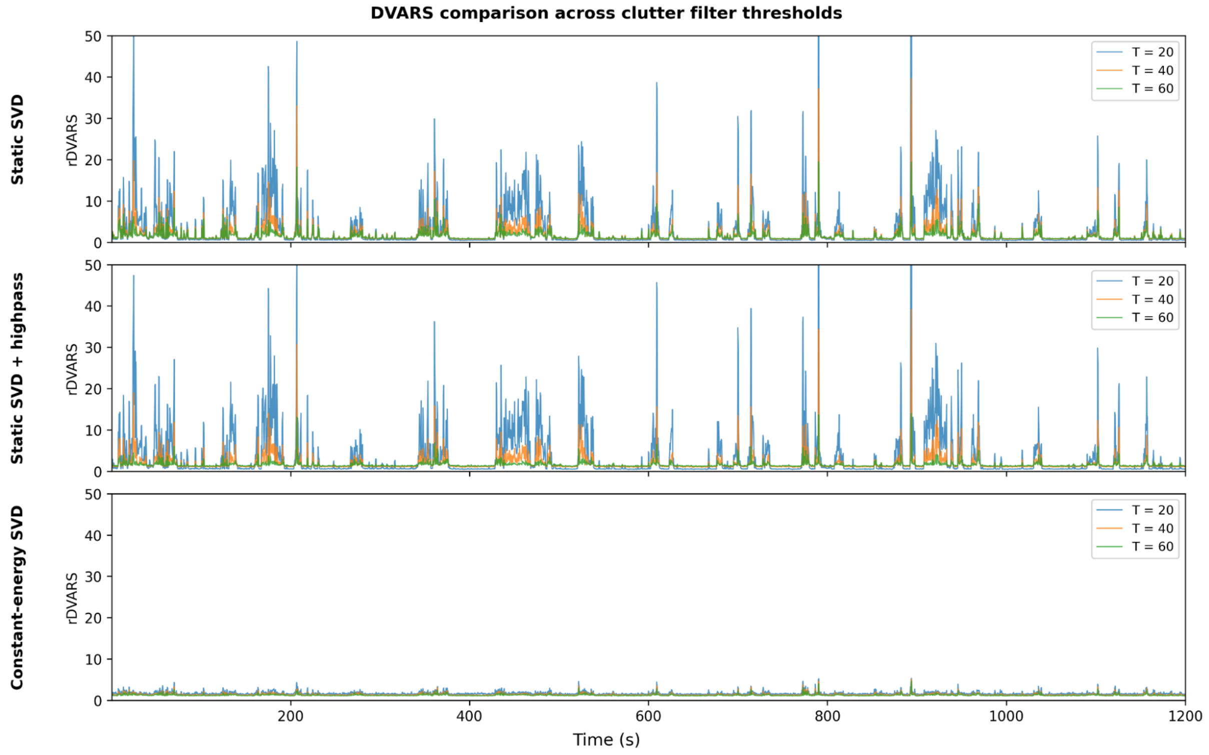

Figure S8: Constant-energy constraints normalize global variance across clutter filter thresholds. Comparison of rDVARS time courses across three SVD thresholds (20, 40, and 60) for the three different filtering strategies used in the standard strategy and paradigms 1 & 2. (Top & middle) Standard Static SVD strategies exhibit high sensitivity to parameter selection; lower thresholds allow higher baseline energy, resulting in large spikes in global variance. (Bottom) The Constant-energy SVD strategy normalizes this variance, keeping it more stable and comparable across all thresholds.
